# AHL-lactonase expression in three marine emerging pathogenic *Vibrio* spp. reduces virulence and mortality in brine shrimp (*Artemia salina*) and Manila clam (*Venerupis philippinarum*)

**DOI:** 10.1371/journal.pone.0195176

**Published:** 2018-04-17

**Authors:** Marta Torres, José Carlos Reina, Juan Carlos Fuentes-Monteverde, Gerardo Fernández, Jaime Rodríguez, Carlos Jiménez, Inmaculada Llamas

**Affiliations:** 1 Department of Microbiology, Faculty of Pharmacy, University of Granada, Granada, Spain; 2 Institute of Biotechnology, Biomedical Research Center (CIBM), University of Granada, Granada, Spain; 3 Department of Chemistry, Faculty of Sciences and Center for Advanced Scientific Research (CICA), University of A Coruña, A Coruña, Spain; 4 Research Support Service (SAI), Central Services (ESCI) University of A Coruña, A Coruña, Spain; Universita degli Studi Roma Tre, ITALY

## Abstract

Bacterial infectious diseases produced by *Vibrio* are the main cause of economic losses in aquaculture. During recent years it has been shown that the expression of virulence genes in some *Vibrio* species is controlled by a population-density dependent gene-expression mechanism known as quorum sensing (QS), which is mediated by the diffusion of signal molecules such as *N*-acylhomoserine lactones (AHLs). QS disruption, especially the enzymatic degradation of signalling molecules, known as quorum quenching (QQ), is one of the novel therapeutic strategies for the treatment of bacterial infections. In this study, we present the detection of AHLs in 34 marine *Vibrionaceae* strains. Three aquaculture-related pathogenic *Vibrio* strains, *V*. *mediterranei* VibC-Oc-097, *V*. *owensii* VibC-Oc-106 and *V*. *coralliilyticus* VibC-Oc-193 were selected for further studies based on their virulence and high production of AHLs. This is the first report where the signal molecules have been characterized in these emerging marine pathogens and correlated to the expression of virulence factors. Moreover, the results of AHL inactivation in the three selected strains have been confirmed *in vivo* against brine shrimps (*Artemia salina*) and Manila clams (*Venerupis philippinarum*). This research contributes to the development of future therapies based on AHL disruption, the most promising alternatives for fighting infectious diseases in aquaculture.

## Introduction

Outbreaks of bacterial disease are considered as a significant constraint to the development of aquaculture in the world, causing serious economic losses in this sector [1]. Although many of the causative pathogens are still unknown, most of them belong to the family *Vibrionaceae* [2]. Vibriosis is one of the most common diseases in aquaculture, affecting a large number of fishes, mollusks, crustaceans and corals [3,4]. *Vibrio* species, such as *V*. *harveyi*, *V*. *vulnificus*, *V*. *anguillarum*, *V*. *ordalii* and *V*. *parahaemolyticus*, are the major causes of vibriosis and are ubiquitous in global marine environments [5]. To date, other emerging species, such as *V*. *neptunius*, *V*. *mediterranei* and *V*. *coralliilyticus* are recently being related to mortality outbreaks worldwide [6,7].

During the last few years virulence of different pathogenic bacteria has been associated to quorum sensing (QS), a sophisticated mechanism which coordinates gene expression by means of small signal molecules known as autoinducers [8,9]. Amongst others, autoinducers include *N*-acylhomoserine lactones (AHLs) produced by the *Proteobacteria*, oligopeptides produced by the *Firmicutes* and furanosylborate diester (AI-2) which are produced by both *Proteobacteria* and *Firmicutes* and are used for interspecies communication [10,11]. In general terms, the basic mechanism of QS systems begins with a basal expression of a signal molecule (autoinducer). The concentration of the autoinducer increases with cell density, and upon reaching a threshold level, it binds and activates a LuxR-type regulator. The autoinducer LuxR-complex then causes the expression of the autoinducer synthase, thereby establishing a positive feedback loop. The result of this induction is the regulation (either positive or negative) of different target genes, some of which involve the expression of virulence factors and exoenzymes, conjugal DNA transfer, control of plasmid-copy number, production of and susceptibility to antibiotics, biofilm formation and exopolysaccharide production [10,11].

To date, QS systems have been studied in different marine pathogenic species belonging to the genera *Vibrio*, *Edwardsiella*, *Aeromonas*, *Pseudomonas* and *Yersinia*. In most cases, the expression of virulence genes such as exoenzymes or pigment production has been reported to be regulated by QS [12–15]. The majority of these studies have focused on the genus *Vibrio*, the main etiological agent of aquatic-related diseases [15–18] and responsible for mass mortalities in a great number of bivalve mollusks [6,19–21]. For instance, AHL-type autoinducers control the production of metalloprotease, siderophores and chitinase A amongst other virulence factors in *Vibrio campbellii* [22–24]; as well as biofilm formation and metalloprotease and siderophore production in *V*. *anguillarum* [13,25].

Besides the ability to produce and use AHL-based communication systems, the ability to interfere with QS systems or to degrade AHLs has been reported for many marine bacteria [26–30], suggesting that these organisms have developed various mechanisms to interfere with or disrupt these cellular communication systems [31–34]. One of the most promising mechanisms that strongly perturbs or even abolishes QS-regulated functions is related to the production of enzymes capable of degrading the AHL-signal molecules, known as quorum quenching (QQ) [33]. To date, different groups of enzymes have been identified according to the enzymatic mechanism involved: the AHL lactonases (lactone hydrolysis), the AHL acylases (amide hydrolysis) and the AHL oxidases/oxidoreductases (oxidoreduction) [35–37].

Research on QS-dependent pathogenic bacteria is becoming a very important tool for understanding and controlling the diseases they cause, and their inhibition by QQ and other strategies has already proven to be one of the most promising alternatives for fighting infectious diseases in aquaculture [38–40]. To increase the information about QS systems and their role in emerging aquaculture-related pathogenic bacteria, we have analyzed the production of AHL signal molecules in 34 strains isolated from healthy and diseased corals and seawater by a well-diffusion agar-plate assay using the biosensor strains *Agrobacterium tumefaciens* NTL4 (pZLR4) and *Chromobacterium violaceum* CV026. The fingerprints and the molecular structure of the AHLs produced by three selected pathogenic strains were then analyzed and compared by TLC and characterized by HPLC/FT-HRMS. In order to investigate the role of QS in the virulence of the selected *Vibrio* strains, AHL lactonases were expressed in each strain, and different phenotypes were investigated in the AHL-defective transformants in comparison with the wild type strains. Additionally, and considering the economic importance of clam and crustacean production in aquaculture and their susceptibility to vibriosis [41,42], Manila clams (*Venerupis philippinarum*) and brine shrimp (*Artemia salina*) were used as infection models to conduct preliminary *in vivo* assays to evaluate the impact of AHL interference upon the virulence of the three selected *Vibrio* strains.

The information generated in this research contributes to the development of new therapies based on AHL disruption, one of the most innovative and promising alternatives for combating infectious diseases in the aquaculture sector, and suggests that QQ is an appropriate strategy to prevent infections caused by these emerging pathogens.

## Material and methods

### 2.1. Bacterial strains, media and growth conditions

The 34 marine strains tested in this study were previously isolated from *Oculina patagonica* and *Cladocora caespitosa* healthy and diseased corals and from seawater and they were identified according to their 16S rRNA gene (~1,500 bp) (Table 1) [43]. They were routinely cultured at 25°C in marine broth (MB), as well as *Vibrio anguillarum* ATCC 19264^T^ [25]. *Agrobacterium tumefaciens* NTL4 (pZLR4) [44] was cultured at 28°C in Luria Bertani (LB) medium or AB medium supplemented with 80 μg/mL of 5-bromo-4-chloro-3-indolyl-β-D-galactopyranoside (X-gal) when needed [45]. *Chromobacterium violaceum* CV026 [46] was grown at 28°C in LB medium. In the laboratory, stocks of the bacterial strains were maintained at -80°C with a cryopreservative.

**Table 1:** Detection of AHLs produced by marine *Vibrionaceae* strains detected by well-diffusion agar-plate assay.

Strain	Phylogenetically most related species	Isolation source	NTL4	CV026
Vcl-20	*Vibrio comitans*	*Cladocora caespitosa* (diseased)	+	-
VibC-Oc-021	*V*. *splendidus*	*Oculina patagonica* (healthy)	-	-
VibC-Oc-024	*V*. *gigantis*	*O*. *patagonica* (healthy)	-	-
VibC-Oc-027	*V*. *gigantis*	*O*. *patagonica* (healthy)	+	-
VibC-Oc-044	*V*. *comitans*	*O*. *patagonica* (healthy)	+	-
VibC-Oc-062	*V*. *breoganii*	*O*. *patagonica* (healthy)	-	-
VibC-Oc-063	*V*. *hepatarius*	*O*. *patagonica* (healthy)	+	-
VibC-Oc-068	*V*. *coralliilyticus*	*O*. *patagonica* (diseased)	+	-
VibC-Oc-071	*V*. *hepatarius*	*O*. *patagonica* (healthy)	+	-
VibC-Oc-079	*V*. *fortis*	*O*. *patagonica* (healthy)	+	-
Vcl-080	*V*. *tubiashii*	*C*. *caespitosa* (diseased)	+	-
VibC-Oc-082	*V*. *maritimus*	*O*. *patagonica* (healthy)	++	++
VibC-Oc-086	*Photobacterium lutimaris*	Seawater	-	-
VibC-Oc-089	*P*. *phosphoreum*	Seawater	+	-
Voc-097	*V*. *maritimus*	*O*. *patagonica* (diseased)	+++	-
VibC-Oc-097	*V*. *mediterranei*	*O*. *patagonica* (diseased)	+++	+
VibC-Oc-106	*V*. *owensii*	Seawater	++	++
VibC-Oc-109	*V*. *fortis*	*O*. *patagonica* (healthy)	-	-
VibC-Oc-110	*V*. *rotiferianus*	Seawater	+	-
VibC-Oc-112	*V*. *alginolyticus*	*O*. *patagonica* (healthy)	+	-
Vcl-121	*V*. *campbellii*	*C*. *caespitosa* (diseased)	+	-
Voc-130	*P*. *rosenbergii*	*O*. *patagonica* (healthy)	+	-
Voc-170	*Listonella pelagia*	*O*. *patagonica* (diseased)	-	+
Vcl-178	*V*. *ponticus*	*C*. *caespitosa* (diseased)	+	-
VibC-Oc-193	*V*. *coralliilyticus*	*O*. *patagonica* (diseased)	++	-
Vcl-234	*V*. *breoganii*	*C*. *caespitosa* (diseased)	+	-
Vcl-245	*V*. *comitans*	*C*. *caespitosa* (diseased)	+	-
Vcl-258	*V*. *coralliilyticus*	*C*. *caespitosa* (diseased)	+	-
Voc-264	*V*. *natriegens*	*O*. *patagonica* (healthy)	+	-
Voc-271	*V*. *natriegens*	*O*. *patagonica* (healthy)	+	-
Voc-273	*V*. *parahaemolyticus*	*O*. *patagonica* (healthy)	+	-
Voc-277	*V*. *harveyi*	*O*. *patagonica* (healthy)	+	-
Voc-279	*Vibrio sp*. BWDY-66	*O*. *patagonica* (healthy)	+	-
Voc-1146	*V*. *mediterraneii*	*O*. *patagonica* (diseased)	-	-

++ strong activation, + medium activation and - no activation of the biosensors *Chromobacterium violaceum* CV026 and *Agrobacterium tumefaciens* NTL4 (pZLR4).

When required, antibiotics were used at the following final concentrations: kanamycin (Km) 50 μg/mL, gentamicin (Gm) 50 μg/mL and tetracycline (Tc) 10 and 75 μg/mL.

### 2.2. Ethical statement

This study was carried out following the fundamental ethical principles with regard to the use of *Artemia salina* and *Venerupis philippinarum*. According to the European normative that regulates minimum fishing size of (Council Regulation No. 1967/2006), only clams of commercial size with minimum length of 25 mm were bought at the local market for this study. *Artemia salina* and *Venerupis philippinarum* are not considered endangered or protected species in any European or international species catalogue, including the CITES list (www.cites.org).

### 2.3. Screening for AHL activity by a well-diffusion agar-plate assay

The detection of AHLs was done as previously described in our laboratory [29,30]. The 34 bacterial strains were grown in 5 mL of MB at 25°C until the early stationary phase (OD_600_ 2.8). An overnight culture of one of the biosensor strains [*C*. *violaceum* CV026 or *A*. *tumefaciens* NTL4 (pZLR4)] was diluted to 1:10 in 5 mL of LB 0.7% (w/v) agar and poured respectively onto AB medium supplemented with X-gal and LB agar plates. Then 6-mm-diameter wells were hollowed in the medium with the back of a sterile Pasteur pipette and 100 μL-aliquots of each culture were loaded in the wells. The plates were incubated at 28°C for 24 h to check for the appearance of a coloured halo around the wells. *V*. *anguillarum* ATCC 19264^T^ was used as positive control and MB medium as a negative control.

### 2.4. AHL extraction and thin-layer chromatography analysis

To extract AHL molecules, 30 mL cultures of the three selected bacteria (*Vibrio mediterranei* VibC-Oc-097, *V*. *owensii* VibC-Oc-106 and *V*. *coralliilyticus* VibC-Oc-193) were grown until the early stationary phase (OD_600_ 2.8). Then, the entire cultures were extracted twice with equal volumes of dichloromethane [47,48]. The extracts were dried and suspended in 100 μL of 70% (v/v) methanol.

To characterize the AHLs produced by each strain, the samples were analyzed by thin-layer chromatography (TLC). 25 μL of each AHL extract and standards were spotted onto a TLC plate (Partisil KC18 Whatman 20 × 20 cm) and developed with 60% (v/v) methanol. After migration, the plates were air-dried and overlaid with top agar containing the appropriate biosensor and incubated at 28°C for 24 h [47,48].

### 2.5. AHL identification by high performance liquid chromatography-Fourier transform-high resolution mass spectrometry

To identify the AHLs produced by the selected *Vibrio* strains by HPLC/FT-HRMS, a litre of whole culture of each strain was grown in MB medium to stationary phase (OD_600_ 2.8) and extracted twice with an equal volume of dichloromethane, evaporated and resuspended in CH_3_CN [47,48].

For the analysis, each extract was purified by solid phase extraction [49] and analyzed by high-performance liquid chromatography coupled with Fourier-transform and high resolution mass spectrometry (HPLC/FT-HRMS) [50,51] using full scan (FS) and selected ion monitoring (SIM) modes in a Thermo Scientific Accela LC system coupled to a LTQ Orbitrap. The software Xcalibur 3.0 was used for data processing in both cases. For the HPLC/FT-HRMS FS mode analysis, the extract was passed through a 0.22 μm PTFE syringe filter before injection and 10 μl of each filtered solution was analyzed (mass range from m/z 50 to 500). Analyses were carried out using an Atlantis C_18_ column (100 mm × 4.6 mm, 5 μm); and a gradient mixture of CH_3_CN (solvent A) and H_2_O (solvent B) containing 0.1% (v/v) formic acid at flow rate of 350 μL/min (S1 Table). In the case of the analysis by HPLC/FT-HRMS SIM mode, each extract was passed through an Oasis HLB cartridge, which had previously been conditioned and equilibrated with 500 μL of CH_3_CN and 500 μL of H_2_O. Then, two fractions were eluted with 500 μL of CH_3_CN and H_2_O mixtures (1:1 and 1:0), and 10 μL of the fraction eluted with a mixture of CH_3_CN/H_2_O (1:0) was analyzed. In this case the analysis was conducted in a Scharlau KromaPhase C_18_ column (150 mm × 4.6 mm, 5 μm) using again a gradient mixture of CH_3_CN (solvent A) and H_2_O (solvent B), containing 0.1% (v/v) formic acid at flow rate of 80 μL/min (S1 Table).

Precision and accuracy were calculated from the relative standard deviation of the replicates (<15%) and by direct comparison of mean measured levels of spiked analytes with expected concentrations for unextracted standards, respectively. The external standard method was used for quantification. LC-MS/MS peak areas were calculated and used to build calibration curves of peak area ratio against analyte concentration using unweighted linear regression analysis (S1 Fig and S2 Table). The lower limit of quantification was defined as the concentration at which a signal/noise ratio of 10:1 was achieved.

### 2.6. Expression of AHL-lactonase genes in *Vibrio* spp

The selected *Vibrio* strains (*V*. *mediterranei* VibC-Oc-097, *V*. *owensii* VibC-Oc-106 and *V*. *coralliilyticus* VibC-Oc-193) were transformed by electroporation [52] with the recombinant plasmids pME6010::*attM*, pME6010::*aiiA*, pME6010::*hqiA* (coding each for an AHL-lactonase gene) [53–55] and with the empty plasmid pME6010 [56] (Tc^R^). Competent cells were obtained after some modifications of the classical protocol [57]. Briefly, 25 mL of overnight cultures of each strain were centrifuged and cells were washed twice and resuspended in sucrose 300 mM (50 mL, 25 mL and 500 μL respectively) at 4°C. Then, 40 ng of each plasmid was transformed into 40 μL of each *Vibrio* competent cells by electroporation [52].

The transformants were confirmed by PCR amplification of the corresponding AHL-lactonase gene. The plasmid DNA of each transformant was extracted by the alkaline lysis protocol [58] and used as the template for the PCR amplification. A specific primer set for each AHL-lactonase gene was used [53–55]: *aiiA* forward 5’-ATGAAGAAAATGAACCATGGA-3’ and *aiiaA* reverse 5’-CTATACAAGCAGTCCAAAAGCC-3’, *attM* forward 5’-GACGCAATGAAACAGAGCCG-3’ and *attM* reverse 5’- AAGAGCGACCTGAACGAAGC-3’ and *hqiA*-T forward 5′-ATGAGTGAAATCACGTTGGC-3′ and *hqiA*-T reverse 5′-CTTTACCCGAAGGATCGTAA-3′.

To determine AHL production in each transformant, AHL molecules were extracted [47,48] from 5 mL-cultures of each *Vibrio* strain. Then, 10 μL of the crude extracts were added to 5-mm sterile disks placed onto AB medium supplemented with 80 μg/mL X-gal with an overlay of *A*. *tumefaciens* NTL4 (pZLR4), made as previously explained. The plates were incubated overnight at 28°C to allow the biosensor to grow and surround the paper disks with blue haloes.

### 2.7. Phenotypic analyses

10 μL of the wild type *Vibrio* and its transformant cultures (harbouring a plasmid with or without an AHL lactonase) (DO_600_ 2.2) were spotted on different media. Proteolytic activity was detected in casein medium [59]. Hemolytic activity was determined in Columbia agar supplemented with 5% (w/v) sheep blood [59]. Chitinase activity was assayed in marine broth supplemented with 1% (w/v) colloidal chitin [60]. DNase activity was tested in DNase agar medium [61]. Lipase activity was determined in marine agar (MA) supplemented with 1% (v/v) Tween 20 or Tween 80 [62]. Amylase activity was assayed in MA added with 1% (w/v) starch [59]. Gelatinase activity was measured in MA supplemented with 1% (v/v) gelatin [63]. The hydrolysis of esculin was tested in MA added with 1% (w/v) esculin and 0.05% (w/v) ferric chloride[64]. The swimming motility test was conducted in MB supplemented with 0.3% (w/v) agar [65], while swarming motility was assayed in MB supplemented with 0.5% (w/v) agar [66]. Acid phosphatase activity was detected in PVK medium [67] and alkaline phosphatase was evaluated in MA supplemented with phenolphthalein phosphate 0.01% (w/v) [68]. In all these media the results were acquired by measuring haloes around the spotted area after a 7-day incubation, excluding the motility tests where growth due to the migration of cells away from the inoculation site was measured after 20 h.

### 2.8. *In vivo* assays

The virulence of the wild type *Vibrio* strains and their transformants (harbouring pME6010 with or without *attM*, *aiiA* and *hqiA*) were preliminarily tested *in vivo* in *Artemia salina* (brine shrimp) nauplii. Additionally, the virulence of *Vibrio coralliilyticus* VibC-Oc-193 was evaluated in *Venerupis philippinarum* (Manila clams) obtained from the local market.

In the case of the brine shrimp evaluation test, the hatching cysts of *Artemia salina* (JBL Artemio Pur) wereobtained following the manufacturer instructions using filtered and autoclaved seawater (SFSW) (salinity 36 g/L, 20°C, pH 7.3). The virulence test was performed as described in the protocol by Ha et al. [69], but with minor modifications. After hatching, 20 brine shrimp nauplii were transferred into a Petri dish (35 × 10 mm) containing 20 mL of SFSW. Then, the bacterial cells were washed twice in SFSW and added in a final concentration of 10^6^ CFU/mL (bacterial concentration was determined by the plate counting method on TCBS) in order to infect the brine shrimps in each condition, and incubated at 25°C for 3 days. As a control, the same volume of SFSW was added to the shrimp larvae and incubated in equal conditions. The survival of the shrimps (detected by the loss of their motility) was scored daily after the addition of bacteria.

The evaluation of virulence against Manila clams was conducted following the methodology described by Drummond et al. [70]. Cultures of *Vibrio* spp. were centrifuged and pellets were washed twice and resuspended in the same volume of SFSW. Eight 25-mm of healthy adult Manila clams were placed in separate empty aquariums, each forming a single non-overlapping layer. 100 mL of SFSW were added to each tank to cover all clams. The bacterial suspension was added to each aquarium, giving a final concentration of 10^6^ CFU/mL, and mixed to ensure even distribution of bacteria amongst the clams. The clams were left undisturbed for 1 h, while they were monitored in order to verify that their shells were open and that they were actively filter-feeding, thereby facilitating the uptake of bacteria. The water was then removed and the clams remained out of water for30 min in order to encourage closure of the valves and the subsequent incorporation of the bacterium within the pallial cavity. In separate control aquariums, control clams were maintained and treated as above, but without the addition of the bacterial suspension. After the infection, the clams were transferred to aerated tanks each containing 4 L of SFSW. Mortalities (detected by the irreversible relax of the adductor muscle and wide opening of the shell valves) were counted during 7 days after inoculation.

For plate counts of the number of vibrios in the aquariums in each *in vivo* experiment, 10-fold serial dilutions of seawater from the tanks were prepared in SFSW, plated on TCBS agar and incubated for 24 h at 25°C.

Each Manila clam assay was done in duplicate and brine shrimp tests were done in triplicate. Differences between mortalities in each experiment were statistically analysed using an ANOVA test (P<0.05) and a Tukey test using R.

## Results

### 3.1. Production of AHLs in the *Vibrionaceae* strains

The synthesis of AHL-type QS signal molecules has been investigated in 34 marine strains (mainly *Vibrio* spp.) isolated from *Oculina patagonica* and *Cladocora caespitosa* corals and from seawater by using a well-diffusion agar-plate assay. The biosensor strains used, *Agrobacterium tumefaciens* NTL4 (pZLR4) and *Chromobacterium violaceum* CV026, developed a blue and purple colour respectively in the presence of exogenous AHLs. These experiments were carried out in triplicate and the same results were obtained. It was found that 27 strains activated *A*. *tumefaciens* NTL4 (pZLR4) and four bacteria induced the purple pigment formation of *C*. *violaceum* CV026 (Table 1).

**Fig 1:**
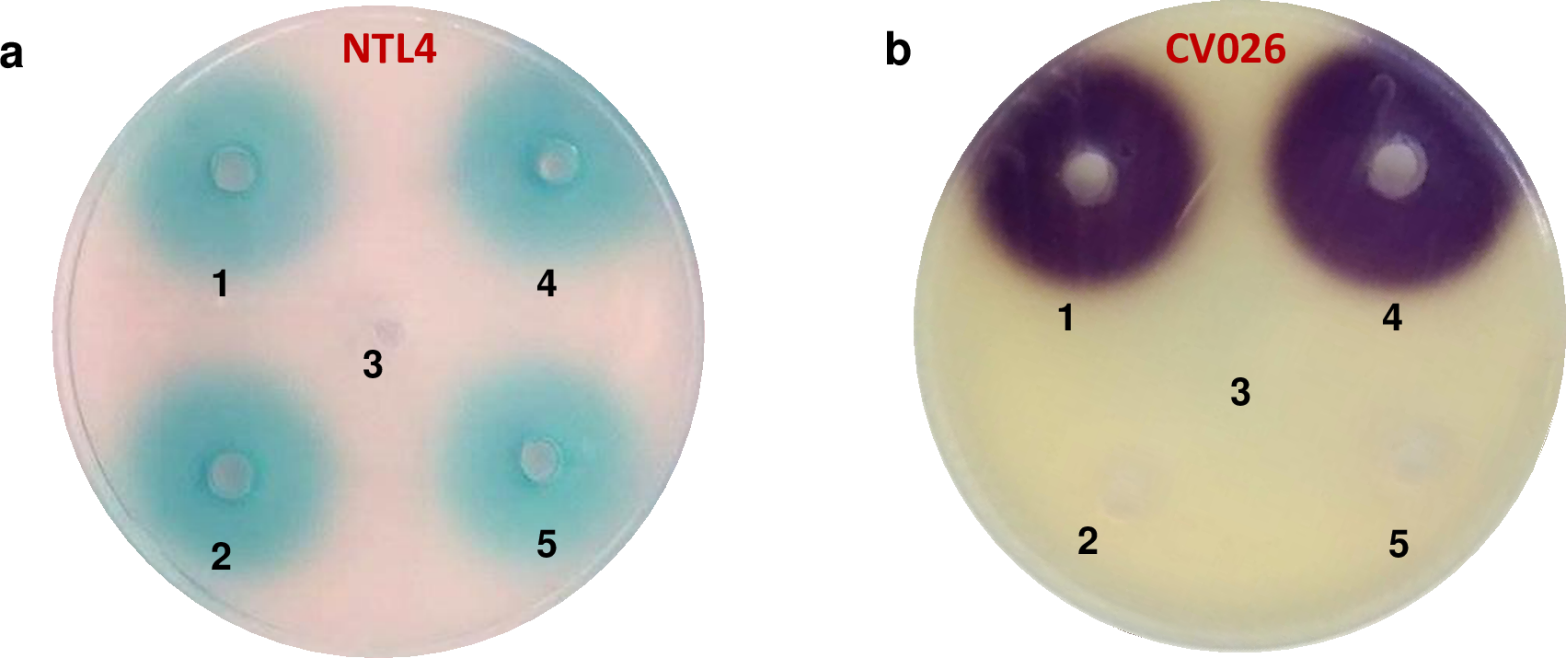
Well-diffusion agar-plate assay to detect AHL production by *Vibrio* spp. 100 μL aliquots of each culture were placed in each well: *Vibrio owensii* VibC-Oc-106 (1), *V*. *coralliilyticus* VibC-Oc-193 (2), *V*. *mediterranei* VibC-Oc-097 (5), *V*. *anguillarum* ATCC 19264^T^ (positive control) (4) and MB (negative control) (3). The biosensors strains used were *Agrobacterium tumefaciens* NTL4 (pZLR4) (a) and *Chromobacterium violaceum* CV026 (b).

Three strains (*Vibrio mediterranei* VibC-Oc-097, *V*. *owensii* VibC-Oc-106 and *V*. *coralliilyticus* VibC-Oc-193) were chosen for further analyses based on the fact that they are considered to be emerging marine pathogenic species and also due to their high virulence and AHL production (Fig 1).

### 3.2. Analysis by TLC of the AHLs produced by the three selected *Vibrio* strains

**Fig 2:**
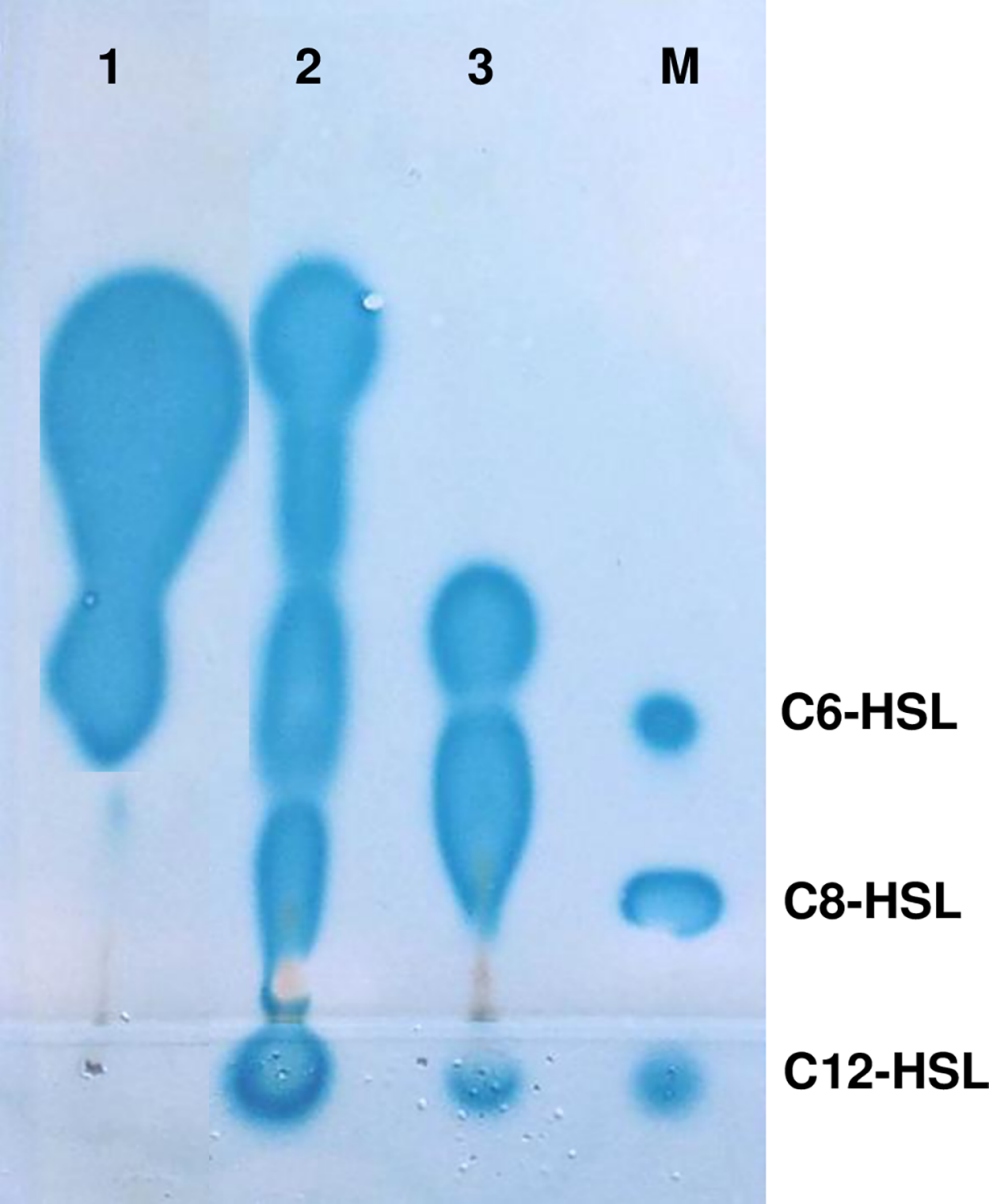
TLC analysis of the AHLs produced by *Vibrio* spp. using the biosensor strain *Agrobacterium tumefaciens* NTL4 (pZLR4). Lane 1, *Vibrio coralliilyticus* VibC-Oc-193; lane 2, *V*. *mediterranei* VibC-Oc-097; lane 3, *V*. *owensii* VibC-Oc-106 and lane M, synthetic AHL standards used as markers: C6-HSL (804 pmol), C8-HSL (31.6 pmol) and C12-HSL (4.8 nmol). Lanes 1 to 3 contain 25 μL of bacterial culture extract.

Culture extracts of the three selected strains were first analyzed by TLC in combination with an overlay of the *A*. *tumefaciens* NTL4 (pZLR4) biosensor. The AHL profiles were different amongst the tested strains, with the detection of at least two active compounds in each strain (Fig 2). In many cases, the signals migrated as tailed spots, indicating that they might be oxo-substituted AHLs. *V*. *mediterranei* VibC-Oc-097 showed the highest diversity of AHLs, with four compounds being produced. Three of them migrated similarly to those of the standards used (C6-HSL, C8-HSL and C12-HSL) and one of them migrated above the C_6_-HSL control. *V*. *coralliilyticus* VibC-Oc-193 produced two spots with motilities above and similar to that of C_6_-HSL. *V*. *owensii* VibC-Oc-106 synthesized at least three detectable signals, one of them chromatographed with a mobility similar to that of C12-HSL and the other two tailed spots migrated above the C6-HSL and C8-HSL standards.

### 3.3. Identification of AHLs by HPLC/FT-HRMS analysis

Signal molecules produced by *V*. *mediterranei* VibC-Oc-097, *V*. *owensii* VibC-Oc-106 and *V*. *coralliilyticus* VibC-Oc-193 were identified and quantified from a large quantity of an early stationary phase culture of each strain.

**Fig 3:**
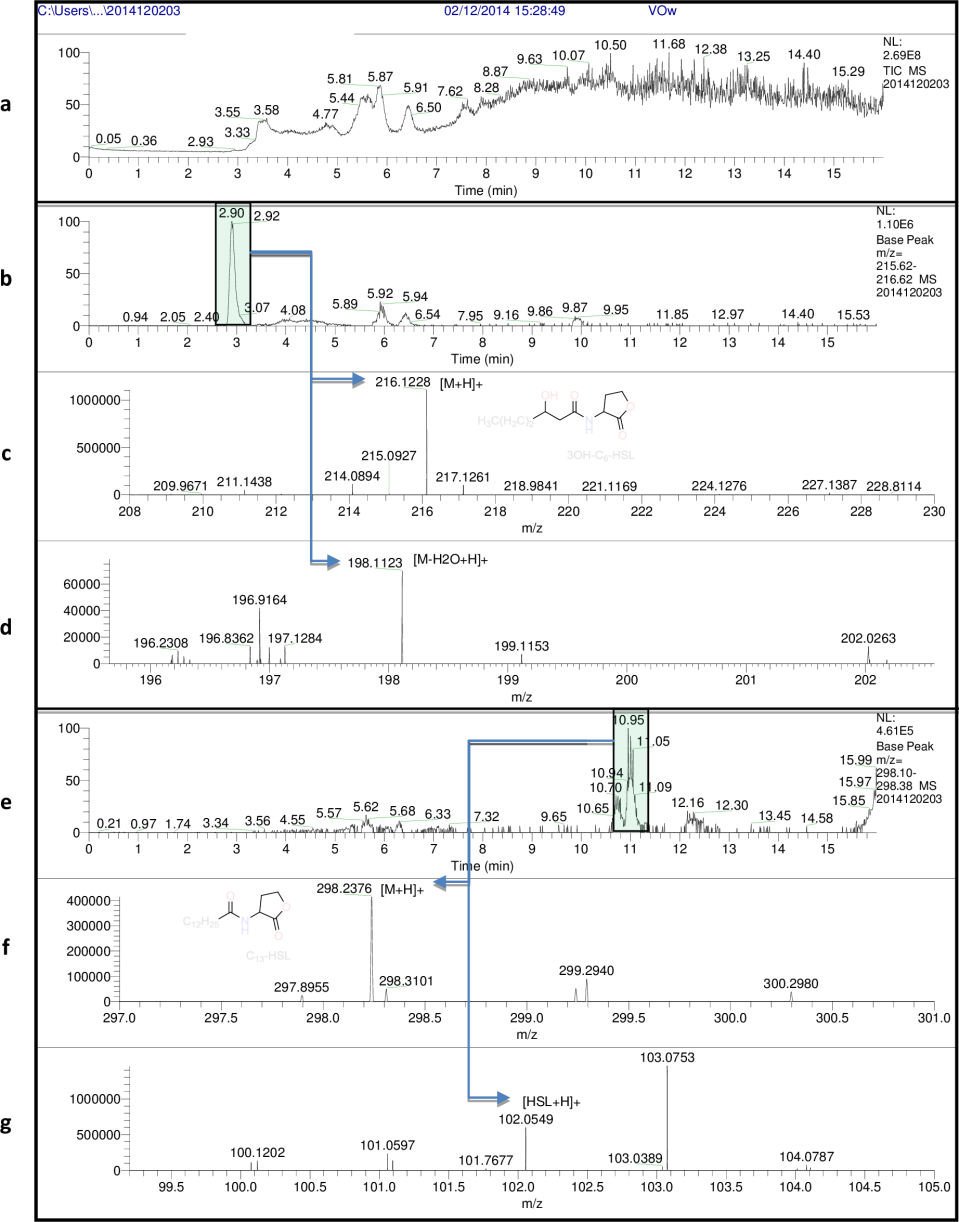
HPLC/FT-HRESIMS (FS mode) experiments for the detection of *N*-acylhomoserine lactones from *Vibrio owensii* VibC-Oc-106. Total ion chromatogram (a). Extracted mass chromatogram (m/z 215.62–216.62) showing the peak at t_*R*_ = 2.90 min (b). Expanded regions of the (+)-HRESIMS of the peak at t_*R*_ = 2.90 min identified as 3-OH-C6-HSL showing the ion peaks [M+H]^+^ at m/z 216.1228 (calc. for C_10_H_18_NO_4_ 216.1230) (c) and [M-H_2_O+H]^+^ at m/z 198.1123 (calc. for C_10_H_16_NO_3_ 198.1125) (d). Extracted mass chromatogram (m/z 298.10–29838) showing the peak at t_*R*_ = 11.00 min (e). Expanded regions of the (+)-HRESIMS of the peak at t_*R*_ = 11.00 min identified as C13-HSL showing the ion peaks [M+H]^+^ at m/z 298.2376 (calc. for C_17_H_32_NO_3_ 298.2377) (f) and [HSL+H]^+^ at m/z 102.0549 (calc. for C_4_H_8_NO_2_ 102.0550) (g).

The identification of AHLs in each strain was initially made by HPLC/FT-HRMS SIM mode analysis using a mixture of twelve AHL standards (Table 2). Detection of further autoinducer molecules was performed by HPLC/FT-HRMS FS mode analysis, which showed the [M+H]^+^ ion peaks of additional AHLs (C9-HSL, C13-HSL, 3-OH-C5-HSL, 3-OH-C6-HSL and 3-OH-C7-HSL) (Table 3). However, the existence of these AHLs could not be confirmed due to the lack of the appropriate standards. Nevertheless, their presence was corroborated by the occurrence in their (+)-high-resolution electrospray ionisation mass spectrometry analysis [(+)-HRESIMS] of the characteristic [102 + H]^+^ ion peak corresponding to the homoserine lactone fragment and the [M+H-18]^+^ and [M+H-101]^+^ ion peaks which correspond to the loss of H_2_O and the HSL moiety (Table 3, S2 Fig). For example, the presence of 3-OH-C6-HSL and C13-HSL in *V*. *owensii* VibC-Oc-106 was corroborated by HPLC/FT-HRMS FS mode analysis through the detection of the corresponding [M-18+H]^+^ ion peak at m/z 198.1123 (calc. for C_10_H_16_NO_3_, 198.1125) for the first HSL and the detection of the [HSL+H]^+^ ion peak at m/z 102.0549 (calc. for C_4_H_8_NO_2_, 102.0550), for the second HSL (Table 3, Fig 3 and S3 Fig). In a similar way, presence of 3-OH-C5-HSL in *V*. *mediterranei* VibC-Oc-097 was confirmed by HPLC/FT-HRMS FS mode analysis through the detection of the corresponding [M-18+H]^+^ ion peak at m/z 184.0968 (calc. for C_9_H_14_NO_3_^+^, 184.0968) (Table 3, S4 Fig).

**Table 2:** AHLs identified by HPLC/FT-HRMS SIM mode analysis in *Vibrio mediterranei* VibC-Oc-097, *V*. *owensii* VibC-Oc-106 and *V*. *coralliilyticus* VibC-Oc-193.

AHL identified			Concentration of AHLs (nmol/L)^a^		
	Ion quantification (m/z)	Retention time^b^ (min)	*V*. *mediterranei* VibC-Oc-097	*V*. *owensii* VibC-Oc-106	*V*. *coralliilyticus* VibC-Oc-193
**C4-HSL**	172.0965	3.82	4.00	nq	3.30
**C6-HSL**	200.1281	3.53	1.33	0.08	nq
**C8-HSL**	228.1594	3.94	0.15	0.11	nq
**C12-HSL**	284.2220	4.87	0.67	4.12	nq
**C16-HSL**	304.2846	4.88	0.64	0.14	0.34
**3-O-C10-HSL**	270.1700	5.63	nq	0.03	nq
**3-O-C12-HSL**	298.2013	6.71	0.02	nq	nq
**3-O-C13-HSL**	312.2169	7.27	1.76	nq	nq
**3-O-C14-HSL**	326.2326	7.81	nq	nq	nq
**3-OH-C10-HSL**	272.1856	5.24	0.36	0.25	0.69
**3-OH-C12-HSL**	300.2169	6.36	8.08	2.77	0.12
**3-OH-C14-HSL**	328.2482	7.53	nq	nq	nq

^a^Calculations were made from the adjusted peak areas from each strain. Data relate to those AHLs which were positively identified against a standard and those that produce a significant quantification ion signal (R>30,000; mass tolerance < 1 ppm).

^b^Retention time of the standards used in the quantification process. nq not quantifiable (limit of detection of AHLs < 0.10 ng/mL).

**Table 3:** AHLs detected by HPLC/FT-HRMS FS mode analysis in *Vibrio mediterranei* VibC-Oc-097 and *Vibrio owensii* VibC-Oc-106.

Microorganism	AHL detected	Retention time (min)	Principal product ions (m/z)	Identity of principal product ions
*V. mediterranei* VibC-Oc-097	3-OH-C5-HSL	6.37	202.1072 184.0968	[M+H]^+^, [M-H_2_O+H]^+^^a^
*V. owensii* VibC-Oc-106	3-OH-C6-HSL	2.90	216.1228, 198.1123	[M+H]^+^, [M- H_2_O+H]^+^
	3-OH-C7-HSL	3.82	230.1381	[M+H]^+^
	C13-HSL	11.00	298.2376 102.0549	[M+H]^+^, [HSL+H]^+^ ^b^

^a^[M-H_2_O +H]^+^ is the result of a loss of water

^b^[HSL+H]^+^ corresponds to the HSL moiety

**Fig 4:**
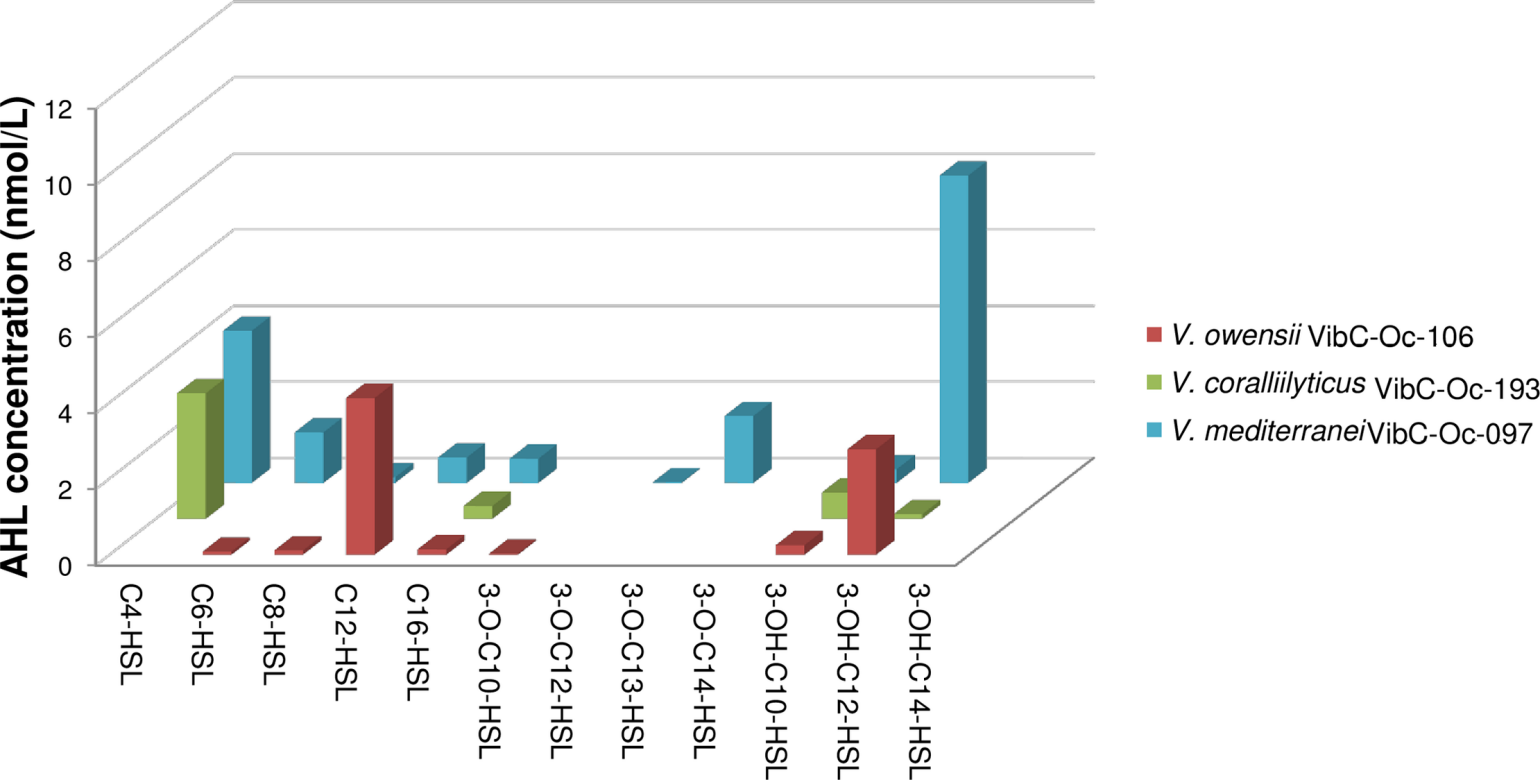
Concentration of the major AHLs quantified in *Vibrio mediterranei* VibC-Oc-097, *V*. *owensii* VibC-Oc-106 and *V*. *coralliilyticus* VibC-Oc-193 using HPLC/FT-HRESIMS (SIM mode). AHL concentration values are shown in nmol/L.

As can be seen in the results obtained, the AHL-profile for each bacterium strain is different (Tables 2 and 3). If the percentage in which the molecules are present in the strains tested is considered, 3-OH-C12-HSL is the most predominant in *V*. *mediterranei* VibC-Oc-097, C4-HSL is the most abundant in *V*. *coralliilyticus* VibC-Oc-193 and C12-HSL is the most commonly produced by *V*. *owensii* VibC-Oc-106. Furthermore, the highest concentration of all the identified AHLs corresponds to 3-OH-C12-HSL in *V*. *mediterranei* VibC-Oc-097 (Table 2, Fig 4).

### 3.4. Selection of AHL-producing defective *Vibrio* strains

In order to evaluate the cellular functions controlled by AHLs, two strategies were used to reduce the AHLs produced by the selected *Vibrio* strains.

**Fig 5:**
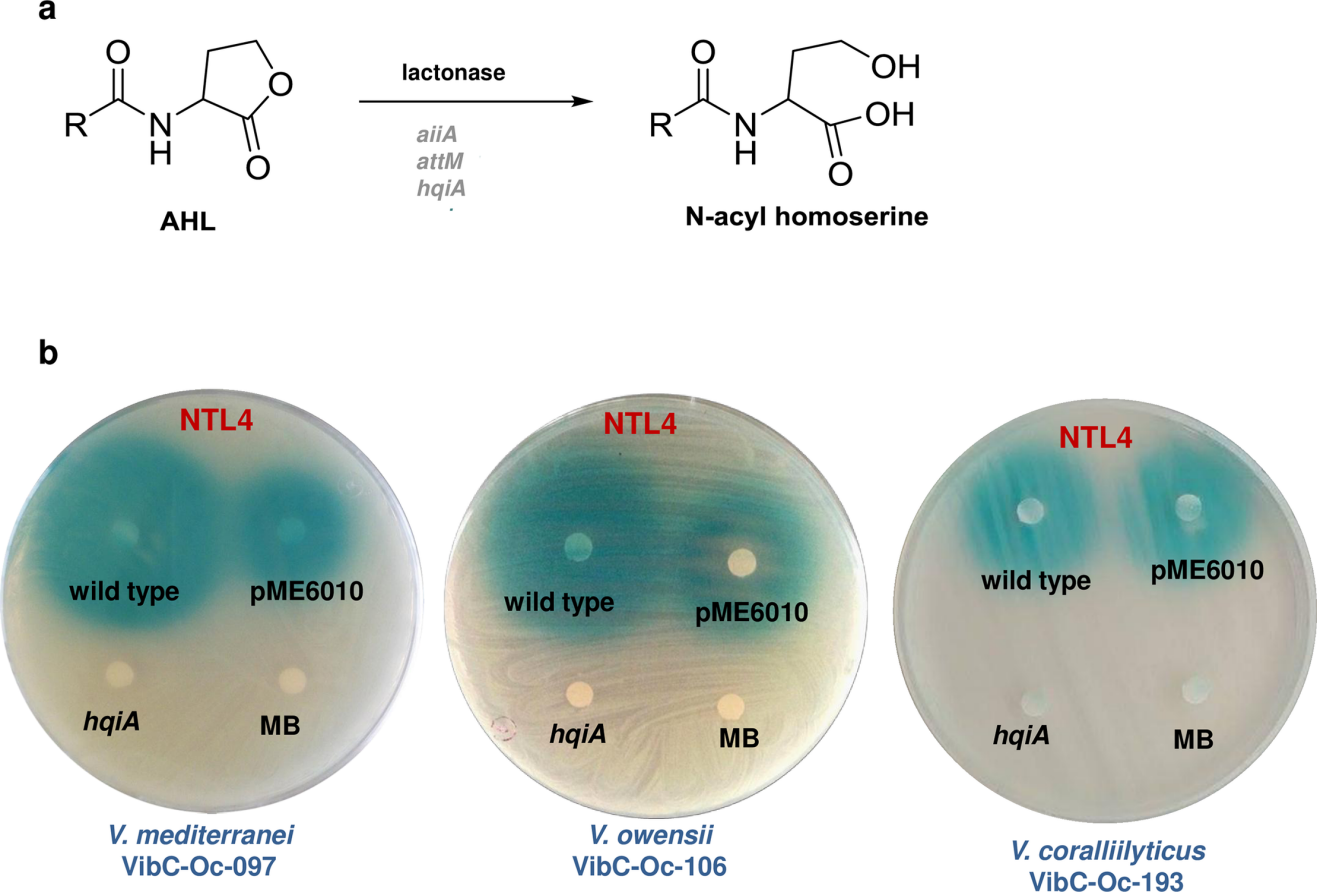
AHL degradation by lactonase-type quorum quenching enzymes. AHL degradation mechanism of the lactonase enzymes AiiA, AttM and HqiA (a). Well-diffusion agar-plate assay to detect AHL production of each wild type *Vibrio* sp. and its transformants (expressing *hqiA* gene) using the biosensor *Agrobacterium tumefaciens* NTL4 (pZLR4). 10 μL-aliquots of each bacterial culture extracts were placed in each sterile disk (b).

As a first strategy, AHL-lactonase genes were expressed in each *Vibrio* strain. Competent cells of the three pathogenic *Vibrio* strains were transformed with recombinant plasmids expressing different AHL lactonase enzymes such as the AiiA, AttM and HqiA, which hydrolyze the lactone ring of AHL signals (Fig 5A). The empty plasmid pME6010 was also transformed into *Vibrio* strains as a negative control. Positive transformants were firstly confirmed by the amplification of the corresponding AHL-lactonase gene (~750–800 bp). Secondly, the transformants containing each of the lactonase genes that had lost the ability to activate the biosensor *A*. *tumefaciens* NTL4 (pZLR4) were selected. In each case, *Vibrio* containing the plasmid pME6010, wild type strains and MB were used as negative controls (Fig 5B).

Additionally, co-cultures of each of the three pathogenic strains *V*. *mediterranei* VibC-Oc-097, *V*. *owensii* VibC-Oc-106 and *V*. *coralliilyticus* VibC-Oc-193 with the AHL-degrading strains PQQ-42 and PQQ-44 [67] were performed in parallel (data not shown). In all the cases, the addition of these QQ strains resulted in a significant reduction of AHLs. None of these strains had a negative effect on the growth of any of the *Vibrio* strains tested (data not shown).

### 3.5. AHL degradation reduces some virulence factors produced by the *Vibrio* spp

**Fig 6:**
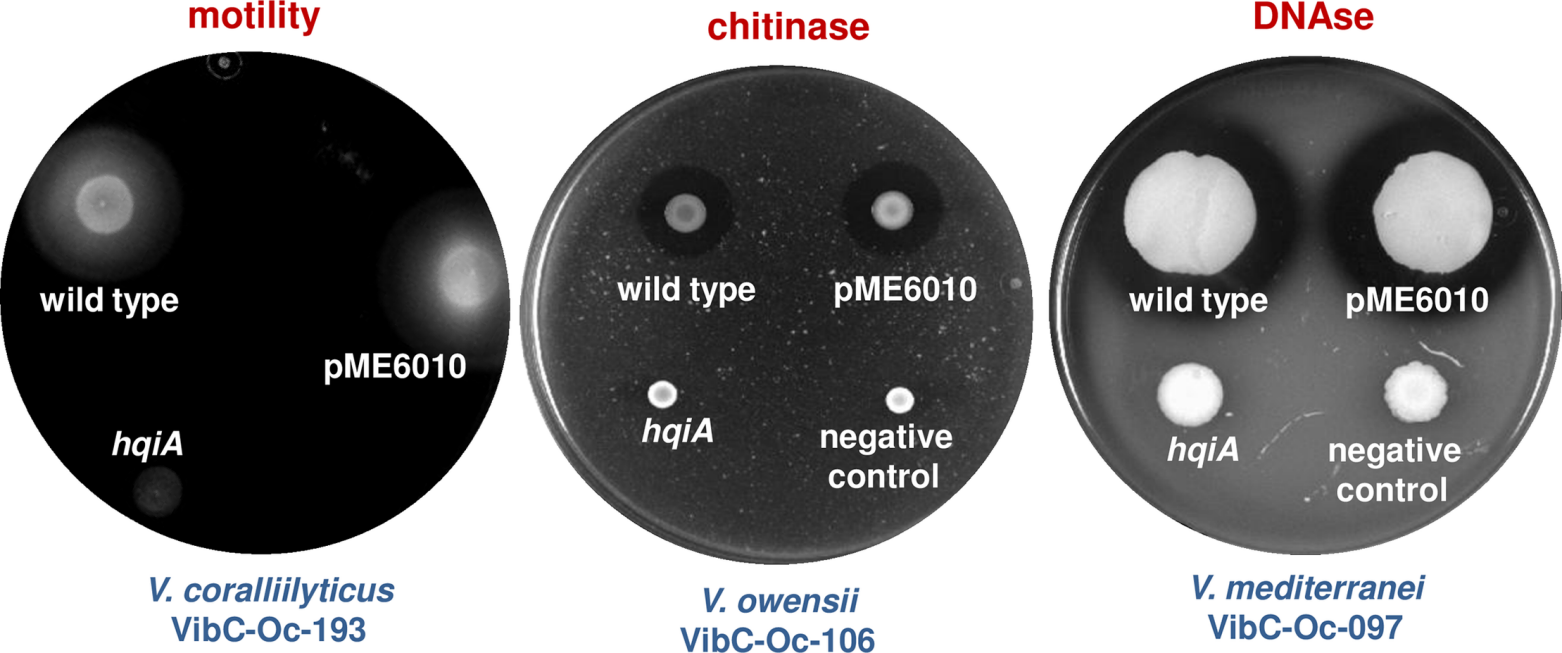
Examples of phenotypes affected by AHL reduction in the three *Vibrio* strains tested.

Different phenotypes were analyzed in the AHL defective *Vibrio* strains (expressing AHL-lactonase genes). The results obtained are the same forthe three lactonases tested, but we only present the outcome after the expression of *hqiA* in Table 4. As shown, the reduction in the accumulation of AHLs in the *Vibrio* strains diminished different phenotypes. For instance, swimming motility was reduced and caseinase and amylase activity was eliminated in *V*. *coralliilyticus* VibC-Oc-193; chitinase and DNAse activities were diminished in *V*. *owensii* VibC-Oc-106 and *V*. *mediterranei* VibC-Oc-097, respectively (Fig 6, Table 4).

**Table 4:** Effect of AHL-degradation upon some of the virulence factors produced by *Vibrio* spp. after the expression of the *hqiA* QQ gene.

Phenotype	*V*. *owensii* VibC-Oc-106			*V*. *mediterranei* VibC-Oc-097			*V*. *coralliilyticus* VibC-Oc-193		
	wild type	pME6010	*hqiA*	wild type	pME6010	*hqiA*	wild type	pME6010	*hqiA*
**AHLs**	++	++	-	++	++	-	++	++	-
**Swimming motility**	++	++	+	++	++	+	++	++	+
**Swarming motility**	++	+	+	++	++	++	++	++	+
**Hemolysis**	++	++	+	++	++	++	++	++	+
**Caseinase**	++	++	++	+	+	+	+++	++	-
**Amylase**	++	++	+	-	-	-	++	++	-
**DNAse**	++	++	+	++	++	+	++	++	++
**Chitinase**	++	++	+	++	++	+	++	++	+++
**Lipase (Tween 20)**	++	++	++	++	++	++	++	++	++
**Lipase (Tween 80)**	++	++	++	++	++	++	++	++	++
**Acid phosphatase**	+	+	+	+	+	+	-	-	-
**Alkaline phosphatase**	++	++	+	++	++	++	++	++	+
**Esculin**	+	+	+	+	+	+	+	+	+
**Gelatinase**	++	++	++	++	++	++	++	++	++

++ strong activity, + medium activity and - no activity after expressing the pME6010 plasmid with and without the *hqiA* QQ gene.

### 3.6. AHL degradation reduces virulence of *Vibrio* spp. on *Artemia salina* and *Venerupis philippinarum*

**Fig 7:**
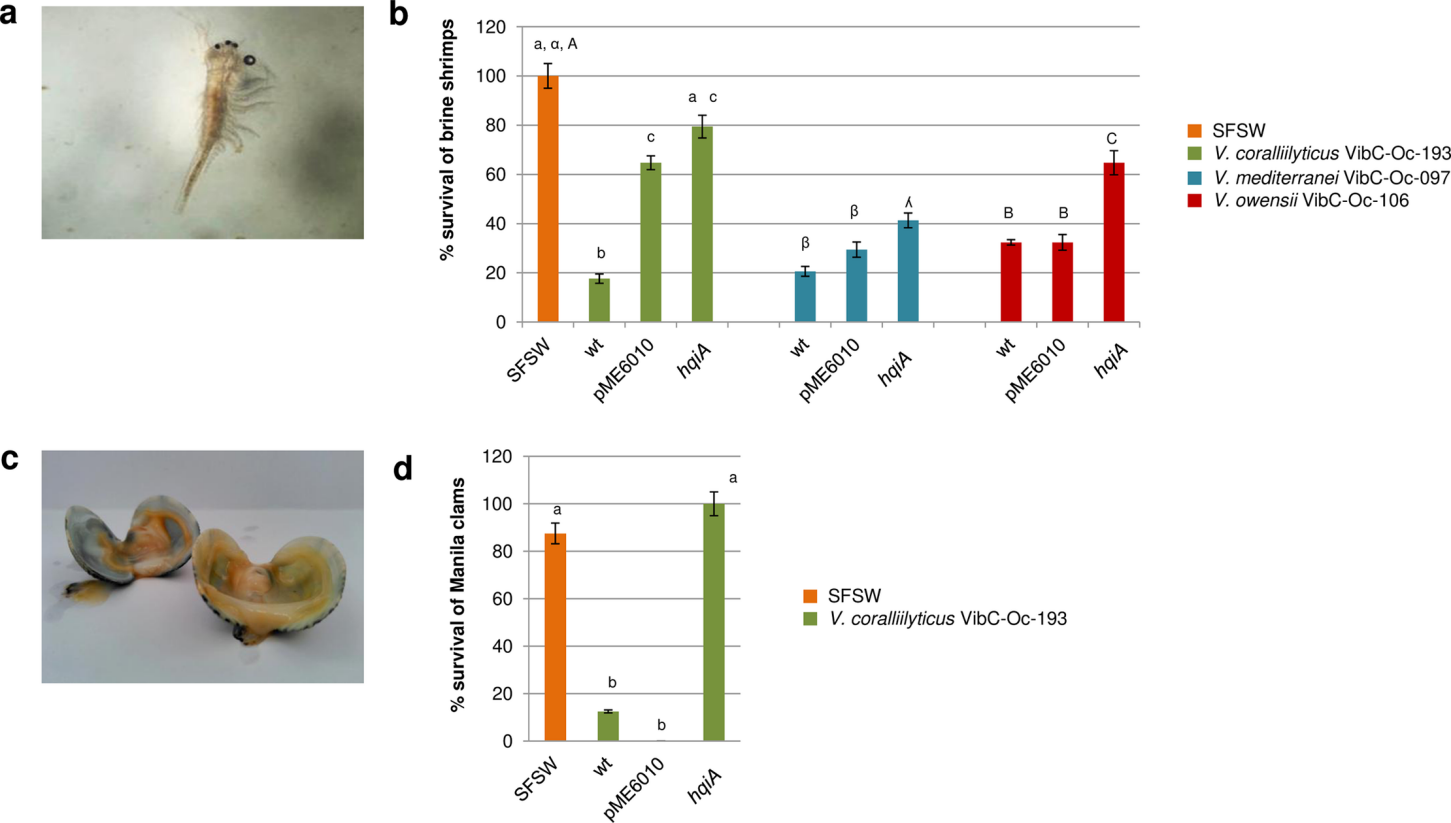
*Artemia salina* and *Venerupis philippinarum in vivo* assays. *Artemia salina* nauplii observed under a magnifying glass (a). Percentage of survival of brine shrimp after inoculation with *V*. *coralliilyticus* VibC-Oc-193, *V*. *mediterranei* VibC-Oc-097, *V*. *owensii* VibC-Oc-106, and their transformants (b). Dead Manila clams showing open valves and decontracted muscle (c). Percentage of survival of Manila clams after the inoculation of *Vibrio coralliilyticus* VibC-Oc-193 and its transformants (d). Error bars represent standard deviations. Different letters above the bars indicate that the values are significantly different (P<0.05).

To test whether the interference of AHL production in *V*. *mediterranei* VibC-Oc-097, *V*. *owensii* VibC-Oc-106 and *V*. *coralliilyticus* VibC-Oc-193 had an impact on virulence, *in vivo* preliminary assays were conducted on *Artemia salina* (brine shrimp) nauplii. Since the expression of some phenotypes and virulence factors were diminished or eliminated after the expression of AHL-lactonase genes, the AHL defective strains harbouring the *hqiA* gene were chosen for these experiments. The loss of motility of the nauplii was observed with a magnifying glass during three days and the results showed that the expression of the *hqiA* AHL-lactonase gene in the tested *Vibrio* spp. significantly reduced the mortality upon *Artemia* nauplii in comparison with the wild type and the pME6010-expressing *Vibrio* strains (Fig 7). Based on the results obtained with the different *Vibrio* species tested, it was noted that *V*. *owensii* VibC-Oc-106 was the most virulent species since it produced a higher mortality on the brine shrimp nauplii.

Additionally, the virulence of *V*. *coralliilyticus* VibC-Oc-193 was also preliminarily tested on *Venerupis philippinarum* (Manila clams), since the other two strains produced a very slow mortality in this invertebrate model and the assay could not be maintained in our laboratory facilities. For each aquarium, death symptoms on the clams (wide open valves and decontracted adductor muscle) were observed during seven days. The results indicated that *V*. *coralliilyticus* VibC-Oc-193 expressing the AHL lactonase gene significantly reduced mortality upon *V*. *philippinarum* in comparison with the controls (Fig 7).

Regarding the number of CFU of *Vibrio* spp. in water in all the experiments conducted, in each case the concentration of bacteria showed similar values throughout the experiment (data not shown).

## Discussion

Marine pathogenic bacteria can completely kill mollusks, fish and coral populations that are cultivated in aquaculture, causing drastic economic losses [71,72]. Even though antibiotics have been used for many years to control infections, the emergence of antibiotic-resistant strains [73,74] and sometimes the inefficacy of the treatments [75] make the situation more and more alarming. Global efforts are needed to search for novel strategies to control pathogens in aquaculture and to promote a responsible use of antibiotics to make the industry more sustainable as well as to maintain a healthy environment [76]. In the case of pathogens which depend on QS to regulate virulence, such as some species of *Vibrio*, the degradation of the AHL signal molecules through QQ could become a good alternative for fighting pathogenicity as pathogen numbers are reduced rather than directly being killed [39,77–79].

To obtain a better understanding of the production of communication signals in aquaculture-related bacteria we have investigated the QS systems in 34 marine strains isolated from healthy and diseased corals and seawater belonging to the genera *Vibrio*, *Photobacterium* and *Listonella* [43]. Although many of these strains have been associated to coral diseases such as bleaching [80–82], most of these diseases have also been related to mollusks, crustaceans and fish. For example, *V*. *coralliilyticus* is now extending its host range to bivalve species such as the hard clam (*Mercenaria mercenaria*), the flat oyster (*Ostrea edulis*), the bay scallop (*Argopecten irradians*), the green-lipped mussel (*Perna canaliculus*) and the naval shipworm (*Teredo navalis*), constituting a serious threat for the bivalve industry, being one of the most important emerging pathogens responsible for larval mortality detected in bivalve hatcheries in France, New Zealand and the United States [6,83,84]. Other species, such as *V*. *owensii* and *V*. *mediterranei* have also been associated to other organisms apart from coral: crustaceans such as the ornate spiny lobster (*Panulirus ornatus*) [85,86] and mollusks such as the blue mussel (*Mytilus edulis*) or the Manila clam (*Venerupis philippinarum*) [87,88]. Nevertheless, the misidentification of several *V*. *coralliilyticus* such as *V*. *tubiashii* and *V*. *neptunius*, and the reclassification of *V*. *shiloi* as *V*. *mediterranei* and of *V*. *communis* as *V*. *owensii* [83,89–91] has complicated the role of these pathogens in fish and mollusk mortality and as the potential etiological agents involved in aquaculture-related diseases.

Nowadays, several methods have been described to easily detect the production of AHLs, such as the "cross-streak" method [92] or the double-layer microplate high-throughput assay that has been performed to test a high number of isolates [16]. In this study we have chosen a well-diffusion agar-plate assay and two biosensor strains based on previous experience in our laboratory [29,30,47]. The biosensors used were *Chromobacterium violaceum* CV026, which responds to exogenously added short-chain AHLs (C4-HSL and C6-HSL), and *Agrobacterium tumefaciens* NTL4 (pZLR4), a sensitive, broad-spectrum AHL-responsive reporter. Our results indicate that four strains induce the biosensor CV026 and 27 out of the 34 bacteria synthesized signal molecules to activate NTL4. In a previous study, Yang et al.[15] have also found that a high number of bacteria (21 out of 25 strains) belonging to the family *Vibrionaceae* induced the biosensor *A*. *tumefaciens*. To date, the use of biosensors that are able to detect the production of AHLs has been very useful for the identification of a large number of AHL-based QS systems [16,93,94].

Additionally, the use of biosensor microorganisms in combination with TLC provides a simple and rapid way of determining the number and nature of the AHLs produced by a particular strain [44]. Based on these techniques some authors have shown the production of AHLs in different strains of the family *Vibrionaceae* [15,94–96].

In this study, we have carried out an in-depth characterization of the AHL signal molecules of three *Vibrio* strains selected by the analysis of their AHL profiles and the virulence capacity of the species to which they are related: *V*. *mediterranei* VibC-Oc-097, *V*. *owensii* VibC-Oc-106 and *V*. *coralliilyticus* VibC-Oc-193. By using a TLC analysis, our results indicate the existence of a high AHL diversity amongst the three species, as other authors have previously reported for other species [15,16].

Since the TLC technique only provides limited information concerning the nature of signal compounds present in the extracts, HPLC/FT-HRMS analyses was essential for determining the structures of these potential signal molecules [50,51]. In this analysis, the whole culture of each strain was used to prepare the extract instead of only using the cell-free supernatant. For this reason, we were able to enhance the extraction of long-chain AHLs, because otherwise their low permeability through the cell membrane limits their extraction and detection [97]. Additionally, no differences were found in the AHL extraction when the supernatant was acidified (data not shown), as suggested by other authors [98]. With respect to the organic solvent needed for the AHL extraction, dichloromethane was used, although no differences were detected when ethyl acetate was used (data not shown). As explained, TLC and HPLC/FT-HRMS analysis showed a high diversity for the production of AHLs molecules by the tested strains, the long-acyl chains AHLs being more predominant in three of the *Vibrio* species analyzed.

The different AHL molecules produced by the three *Vibrio* spp. tested may be the result of the presence of several LuxI synthases acting in a multiple signalling system, as occurs in *Pseudomonas aeruginosa* [99], or the same LuxI synthesizing multiple AHLs based on the intracellular availability of acyl-carrier proteins, as described in *Halomonas anticariensis* [100].

To date, it has been shown that virulence is regulated by QS in many *Vibrio* spp. affecting fish and shellfish. Despite the extensive knowledge about the molecules involved and the biological processes controlled by intercellular communication in some *Vibrio* strains, less is known about the diversity and role of AHLs produced by a wide range of emerging pathogenic species, such as *V*. *coralliilyticus* or *V*. *mediterranei*. The in-depth study of QS is important for a better understanding of their pathogenicity and for the development of future treatments for the diseases, they cause. In a previous study, we demonstrated that AHL-degradation protects *Oculina patagonica* coral from bleaching caused by *V*. *mediterranei* VibC-Oc-097. The addition of the AHL-degrading strain PQQ-42 completely eliminated the AHLs produced by *V*. *mediterranei* VibC-Oc-097, reducing its swimming motility and protease activity, which therefore seem to be regulated by QS systems [30]. AHL degradation in other *Vibrio* species has also helped to understand the role of QS in the regulation of virulence factors, such as biofilm or hemolysin production [16,101]. In this study, the results obtained indicate that the expression of AHL-lactonase genes (*aiiA*, *attM* or *hqiA*) in the pathogenic selected *Vibrio* strains resulted in a significant decrease of AHL accumulation and a drastic reduction of some potential virulence factors, such as the production of protease and chitinase, swimming motility and hemolysis [39,102,103]. Additionally, the AHL defective strains of each selected *Vibrio* produced a significant reduction of the virulence *in vivo* in brine shrimp (*Artemia salina*). In the case of *V*. *coralliilyticus* VibC-Oc-193, the AHL defective strain was significantly less virulent against Manila clams (*Venerupis philippinarum*) when compared with the control, originating a 100% survival, which is the second most important bivalve in aquaculture worldwide with a very important commercial value [40]. Although these results are preliminary, they indicate that the expression of the QQ enzyme HqiA [55] plays an important role in attenuating the virulence in the invertebrate models used, reinforcing the idea that QS is the mechanism involved in the regulation of the virulence of these *Vibrio* strains.

To the best of our knowledge, this is the first report where the QS signal molecules have been characterized and correlated to the expression of virulence factors in the emerging pathogenic species *V*. *owensii*, *V*. *mediterranei* and *V*. *coralliilyticus*. Therefore, this research paves the way to the future development of new therapies based on quorum quenching, an AHL-disruption approach that has already been successfully undertaken for the control of infection in many marine animals.

## Supporting information

S1 Fig Selected ion chromatograms of the standards used for the quantification of AHLs by HPLC/FT-HRMS SIM mode. 800 ng/mL (a) and 1,000 ng/mL (b and c). External calibration curves by adding C4-HSL, C16-HSL, 3-O-C10-HSL, 3-O-C12-HSL, 3-O-C13-HSL 3-OH-C10-HSL, 3-OH-C12-HSL, 3-OH-C13-HSL and 3-OH-C14-HSL.
(DOCX)

S2 Fig Chemical structures of AHLs. General AHL structure (a). Mass spectrometry fragmentation pathway of AHLs (b).
(DOCX)

S3 Fig HPLC/FT-HRESIMS experiments for the detection of *N*-acylhomoserine lactones from *Vibrio owensii* VibC-Oc-106. Total ion chromatogram (a). Extracted mass chromatogram (m/z 230.13–230.14) showing the peak at t_*R*_ = 3.82 min (b). Expanded regions of the (+)-HRESIMS of the peak at t_*R*_ = 3.82 min identified as 3-OH-C7-HSL showing the [M+H]^+^ ion peak at m/z 230.1381 (calc. for C_11_H_20_NO_4_ 230.1387) (c).
(DOCX)

S4 Fig HPLC/FT-HRESIMS experiments for the detection of *N*-acylhomoserine lactones from *Vibrio mediterranei* VibC-Oc-097. Total ion chromatogram (a). Extracted mass chromatogram (*m/z* 202.00–202.22) showing the peak at t_*R*_ = 6.37 min (b). Expanded region of the (+)-HRESIMS of the peak at t_*R*_ = 6.37 min identified as 3-OH-C5-HSL showing the ion peaks [M+H]^+^ at m/z 202.1073 (calc. for C_9_H_16_NO_4_ 202.1074) (c). Expanded region of the (+)-HRESIMS of the peak at t_*R*_ = 6.37 min identified as 3-OH-C5-HSL showing the [M-H_2_O+H]^+^ ion peak at m/z 184.0968 (calc. for C_9_H_14_NO_3_ 184.0968) (d).
(DOCX)

S1 Table Chromatographic profiles used in the HPLC/FT-HRMS FS and SIM mode analyses. (PDF)

S2 Table Data used to calculate the AHL concentration in each *Vibrio* strain by HPLC/FT-HRMS SIM mode analysis. Numbers are counts in the mass spectra.
(PDF)
