# Supplementary material for: AHL-lactonase expression in three marine emerging pathogenic Vibrio spp. reduces virulence and mortality in brine shrimp (Artemia salina) and Manila clam (Venerupis philippinarum)
Source: PLoS One. 2018 Apr 17;13(4):e0195176. doi: 10.1371/journal.pone.0195176 (PMC5903640; doi:10.1371/journal.pone.0195176)
Supplement: S1 Fig — 800 ng/mL (a) and 1,000 ng/mL (b and c). External calibration curves by adding C4-HSL, C16-HSL, 3-O-C10-HSL, 3-O-C12-HSL, 3-O-C13-HSL 3-OH-C10-HSL, 3-OH-C12-HSL, 3-OH-C13-HSL and 3-OH-C14-HSL. (DOCX) [file pone.0195176.s001.docx]

|   **a**  **b**  **c**  **C6-HSL**  **C8-HSL**  **C12-HSL**  m/z = 200.12-200.13  m/z = 228.14-228.16  m/z = 284.21-284.23 |  |
| --- | --- |
|   **C4-HSL**  **3-O-C10-HSL**  **3-O-C12-HSL**  **C16-HSL**  **3-OH-C10-HSL**  **3-OH-C12-HSL**  **3-OH-C13-HSL**  **3-OH-C14-HSL**  **3-O-C13-HSL**  m/z = 272.18-272.19  m/z = 300.21-300.22  m/z = 312.21-312.22  m/z = 328.24-328.25  m/z = 326.23-326.24 |  |
|   m/z = 172.09-172.10  m/z = 304.28-304.29  m/z = 270.16-270.18  m/z = 298.19-298.21 |  |

**Figure S1.** **Selected ion chromatograms of the standards used for the quantification of AHLs by HPLC/FT-HRMS SIM Mode.** 800 ng/mL (a) and 1,000 ng/mL (b and c). External calibration curves by adding C4-HSL, C16-HSL, 3-O-C10-HSL, 3-O-C12-HSL, 3-O-C13-HSL 3-OH-C10-HSL, 3-OH-C12-HSL, 3-OH-C13-HSL and 3-OH-C14-HSL.
