# Supplementary material for: AHL-lactonase expression in three marine emerging pathogenic Vibrio spp. reduces virulence and mortality in brine shrimp (Artemia salina) and Manila clam (Venerupis philippinarum)
Source: PLoS One. 2018 Apr 17;13(4):e0195176. doi: 10.1371/journal.pone.0195176 (PMC5903640; doi:10.1371/journal.pone.0195176)
Supplement: S2 Fig — General AHL structure (a). Mass spectrometry fragmentation pathway of AHLs (b). (DOCX) [file pone.0195176.s002.docx]

**a**

**b**

**Figure S2. Chemical structures of AHLs.** General AHL structure (a). Mass spectrometry fragmentation pathway of AHLs (b).
