# Supplementary material for: AHL-lactonase expression in three marine emerging pathogenic Vibrio spp. reduces virulence and mortality in brine shrimp (Artemia salina) and Manila clam (Venerupis philippinarum)
Source: PLoS One. 2018 Apr 17;13(4):e0195176. doi: 10.1371/journal.pone.0195176 (PMC5903640; doi:10.1371/journal.pone.0195176)
Supplement: S3 Fig — Total ion chromatogram (a). Extracted mass chromatogram (m/z 230.13–230.14) showing the peak at tR = 3.82 min (b). Expanded regions of the (+)-HRESIMS of the peak at tR = 3.82 min identified as 3-OH-C7-HSL showing the [M+H]+ ion peak at m/z 230.1381 (calc. for C11H20NO4 230.1387) (c). (DOCX) [file pone.0195176.s003.docx]

**a**

**b**

**c**

**A**

**B**

**C**

**Figure S3.** **HPLC/FT-HRESIMS experiments for the detection of *N*-acylhomoserine lactones from *Vibrio owensii* VibC-Oc-106.** Total ion chromatogram (a). Extracted mass chromatogram (m/z 230.13-230.14) showing the peak at t*_R_* = 3.82 min (b). Expanded regions of the (+)-HRESIMS of the peak at t*_R_* = 3.82 min identified as 3-OH-C7-HSL showing the [M+H]^+^ ion peak at m/z 230.1381 (calc. for C_11_H_20_NO_4_ 230.1387) (c).
