# Supplementary material for: AHL-lactonase expression in three marine emerging pathogenic Vibrio spp. reduces virulence and mortality in brine shrimp (Artemia salina) and Manila clam (Venerupis philippinarum)
Source: PLoS One. 2018 Apr 17;13(4):e0195176. doi: 10.1371/journal.pone.0195176 (PMC5903640; doi:10.1371/journal.pone.0195176)
Supplement: S4 Fig — Total ion chromatogram (a). Extracted mass chromatogram (m/z 202.00–202.22) showing the peak at tR = 6.37 min (b). Expanded region of the (+)-HRESIMS of the peak at tR = 6.37 min identified as 3-OH-C5-HSL showing the ion peaks [M+H]+ at m/z 202.1073 (calc. for C9H16NO4 202.1074) (c). Expanded region of the (+)-HRESIMS of the peak at tR = 6.37 min identified as 3-OH-C5-HSL showing the [M-H2O+H]+ ion peak at m/z 184.0968 (calc. for C9H14NO3 184.0968) (d). (DOCX) [file pone.0195176.s004.docx]

**

**a**

**b**

**c**

**d**

**A**

**B**

**C**

**D**

**B**

**C**

**D**

**Figure S4. HPLC/FT-HRESIMS experiments for the detection of *N*-acylhomoserine lactones from *Vibrio mediterranei* VibC-Oc-097.** Total ion chromatogram (a). Extracted mass chromatogram (*m/z* 202.00-202.22) showing the peak at t*_R_* = 6.37 min (b). Expanded region of the (+)-HRESIMS of the peak at t*_R_* = 6.37 min identified as 3-OH-C5-HSL showing the ion peaks [M+H]^+^ at m/z 202.1073 (calc. for C_9_H_16_NO_4_ 202.1074) (c). Expanded region of the (+)-HRESIMS of the peak at t*_R_* = 6.37 min identified as 3-OH-C5-HSL showing the [M-H_2_O+H]^+^ ion peak at m/z 184.0968 (calc. for C_9_H_14_NO_3_ 184.0968) (d).

**G**

**L**

**K**

**J**

**I**

**H**

**F**

**D**

**E**
