# Supplementary material for: AHL-lactonase expression in three marine emerging pathogenic Vibrio spp. reduces virulence and mortality in brine shrimp (Artemia salina) and Manila clam (Venerupis philippinarum)
Source: PLoS One. 2018 Apr 17;13(4):e0195176. doi: 10.1371/journal.pone.0195176 (PMC5903640; doi:10.1371/journal.pone.0195176)
Supplement: S1 Table — (PDF) [file pone.0195176.s005.pdf]

Table S1. Chromatographic profiles used in the HPLC/FT-HRMS FS and SIM mode analyses.

Mode	Step	Time (min)	% CH_3CN (0.1% formic acid)	% H_2O (0.1% formic acid)
FS[a]	1	0	30	70
	2	5	80	20
	3	7	95	5
	4	12	95	5
	5	13	30	70
	6	16	30	70
	7	18	90	10
SIM[b]	1	0	0	100
	2	1	0	100
	3	1.5	50	50
	4	5.5	90	10
	5	8	99	1
	6	13	99	1
	7	13.2	0	100
	8	18	0	100

[a]Flow rate of 80 µL/min.
[b]Flow rate of 350 µL/min.
