# Supplementary material for: AHL-lactonase expression in three marine emerging pathogenic Vibrio spp. reduces virulence and mortality in brine shrimp (Artemia salina) and Manila clam (Venerupis philippinarum)
Source: PLoS One. 2018 Apr 17;13(4):e0195176. doi: 10.1371/journal.pone.0195176 (PMC5903640; doi:10.1371/journal.pone.0195176)
Supplement: S2 Table — Numbers are counts in the mass spectra. (PDF) [file pone.0195176.s006.pdf]

Table S2. Data used to calculate the AHL concentration in each *Vibrio* strain by HPLC/FT-HRMS SIM mode analysis. Numbers are counts in the mass spectra.

AHL concentration (ng/mL)	C4-HSL[a]	C6-HSL	C8-HSL	C12-HSL	C16-HSL	3-O-C10-HSL	3-O-C12-HSL	3-O-C13-HSL	3-OH-C10-HSL	3-OH-C12-HSL	3-OH-C13-HSL	3-OH-C14-HSL
**0.4**	16536	-[b]	-	-	-	6005	3952	7699	18541	15989	5414	33127
**4.0**	23347	-	-	-	-	188155	172283	155736	286136	192380	174672	225254
**20.0**	83460	-	-	-	-	927377	1068419	854272	1361511	926104	890182	1253045
**37.5**	-	722787	1675846	7372036	-	-	-	-	-	-	-	-
**75.0**	287128	1764720	3763713	15162081	52376	3332762	3562948	2663970	5212194	3520142	3070748	4141870
**150.0**	560013	3220846	6370386	29729334	-	6830983	7992041	6861302	10042822	6882957	6944537	7962442
**300.0**	1139650	7695713	14674226	56614581	-	12966093	15158010	12917475	17304998	13116674	12983242	14718011
**500.0**	1831893	-	-	-	112751	20978191	23821804	20488901	25799960	20912686	21747024	23458510
**750.0**	-	14289711	24277824	102489159	-	-	-		-	-	-	-
**800.0**	3044747	-	-	-	125248	31351451	36952643	31346502	40525788	33247003	34819034	40391010
**1000.0**	-	18220568	33089439	135380554	-	-	-	-	-	-	-	-

	**Linear fitting for each standard**	
**AHL standard**	**Calibration curve**	**Adjusted R-squared**
**C4-HSL**	y = 3762.05x + 3653.52	R^2 = 0.9994
**C6-HSL**	y = 17948.66x + 734677.94	R^2 = 0.9839
**C8-HSL**	y = 31214.48x + 1.94466E6	R^2 = 0.9763
**C12-HSL**	y = 128310.81x + 8.33816E6	R^2 = 0.9860
**C16-HSL**	y = 108.40x + 42415.45	R^2 = 0.9580
**3-O-C10-HSL**	y = 39673.20x + 401173.9	R^2 = 0.9972
**3-O-C12-HSL**	y = 46463.99x + 350199.5	R^2 = 0.9982
**3-O-C13-HSL**	y = 39650.32x + 245819.0	R^2 = 0.9973
**3-OH-C10-HSL**	y = 50245.13x + 953573.8	R^2 = 0.9946
**3-OH-C12-HSL**	y = 41453.76x + 268668.7	R^2 = 0.9994
**3-OH-C13-HSL**	y = 43499.44x + 23373.51	R^2 = 0.9998
**3-OH-C14-HSL**	y = 49359.17x + 112302.0	R^2 = 0.9977

[a]Columns indicate area peak of the analyte

[b]Missing area peaks indicate that this concentration was not used during calibration process
